# Training and evaluation corpora for the extraction of causal relationships encoded in biological expression language (BEL)

**DOI:** 10.1093/database/baw113

**Published:** 2016-08-20

**Authors:** Juliane Fluck, Sumit Madan, Sam Ansari, Alpha T. Kodamullil, Reagon Karki, Majid Rastegar-Mojarad, Natalie L. Catlett, William Hayes, Justyna Szostak, Julia Hoeng, Manuel Peitsch

**Affiliations:** 1 Fraunhofer Institute for Algorithms and Scientific Computing, Schloss Birlinghoven, Sankt Augustin, Germany; 2 Philip Morris International R&D, Philip Morris Products S.A, Quai Jeanrenaud 5, Neuchâtel, 2000, Switzerland; 3 Department of Health Science Research, Mayo Clinic, Rochester, MN, USA; 4 Selventa, One Alewife Center, Cambridge, MA 02140, USA

## Abstract

Success in extracting biological relationships is mainly dependent on the complexity of the task as well as the availability of high-quality training data. Here, we describe the new corpora in the systems biology modeling language BEL for training and testing biological relationship extraction systems that we prepared for the BioCreative V BEL track. BEL was designed to capture relationships not only between proteins or chemicals, but also complex events such as biological processes or disease states. A BEL nanopub is the smallest unit of information and represents a biological relationship with its provenance. In BEL relationships (called BEL statements), the entities are normalized to defined namespaces mainly derived from public repositories, such as sequence databases, MeSH or publicly available ontologies. In the BEL nanopubs, the BEL statements are associated with citation information and supportive evidence such as a text excerpt. To enable the training of extraction tools, we prepared BEL resources and made them available to the community. We selected a subset of these resources focusing on a reduced set of namespaces, namely, human and mouse genes, ChEBI chemicals, MeSH diseases and GO biological processes, as well as relationship types ‘increases’ and ‘decreases’. The published training corpus contains 11 000 BEL statements from over 6000 supportive text excerpts. For method evaluation, we selected and re-annotated two smaller subcorpora containing 100 text excerpts. For this re-annotation, the inter-annotator agreement was measured by the BEL track evaluation environment and resulted in a maximal *F*-score of 91.18% for full statement agreement. In addition, for a set of 100 BEL statements, we do not only provide the gold standard expert annotations, but also text excerpts pre-selected by two automated systems. Those text excerpts were evaluated and manually annotated as true or false supportive in the course of the BioCreative V BEL track task.

**Database URL:** http://wiki.openbel.org/display/BIOC/Datasets

## Introduction

Published literature remains the largest resource of scientific information and the growth of publications poses a significant challenge in information access and processing. To use this information in the fields of systems biology and systems toxicology, the published data must be converted into a structured format suitable for modeling, reasoning, large-scale querying, and further computational analysis. There is an increasing demand from systems biologists/toxicologists to access such computable network information (1).

However, it is a work-intensive task to manually extract relevant information from primary literature and convert the free text data into structured relationships using controlled vocabularies (2). To simplify the curation task and to reduce the time spent on document triage as well as information extraction, automated support systems must be integrated into curator workflows.

Many databases have already integrated text mining processes into their curation workflows [for an overview, see (3)]. Named entity recognition (NER) tools such as gene and protein name recognition are widely established and used within the database community. Nearly all organism-based databases use NER tools such as Textpresso (4) or ProMiner (5) to identify specific entity classes or assign concept classes (6, 7). PubTator (8) was developed for various manual curation projects and also includes NER. For some entity classes such as biological processes or chemical entities, NER remains challenging (9). BioCreative IV and V have dedicated special tracks for training and evaluation of those entity classes (10–12). There is strong interest in integrating further information extraction processes into curation workflows. The Comparative Toxicogenomics Database, e.g. organized a BioCreative V track to improve and evaluate chemical–disease relation extraction from Medline abstracts (13).

Despite the continuous advancements in biomedical text mining, there is a pressing need to provide more reliable tools for the extraction of relationships suitable for systems biology and toxicology approaches. Large-scale collections of these relationships support researchers in analysing their experimental data on, e.g. diseases and facilitates the identification of critical biomedical entities as therapeutic targets (14–16). However, the lack of training data and evaluation environments remains a major drawback for advancing text mining approaches. There are already a number of corpora for different relationship types available [e.g. drug–drug (17), protein–protein (18), drug–disease interactions (13), gene mutation-disease relationships (19) or disease-phenotype relationships (20)]. Furthermore, a number of assessments such as previous BioCreative or BioNLP tasks addressed relationship extractions in the biomedical domain. For overviews of the BioCreative IV track, we refer to Arighi et al. (21). In reference (22), an overview of the BioNLP 2013 tasks is given. In the pathway curation task of the BioNLP shared task 2013 (23), relationships between chemical compounds and proteins were annotated. The cancer genetics task tackled relationships with diseases and biological processes. However, no normalized entities and no formal language such as BEL were conceptually applied in the previous tasks. In (24), relationships extracted from text are converted into standardized phamacogenomic relationships using the PHARE (PHArmacogenomics RElationship) ontology. Unfortunately, no text corpus is available from this work.

To understand all aspects at multiple levels of relationships spanning from molecular relationships between proteins or chemical entities to relationships with biological processes or diseases, a corpus including those different relationship types is needed.

This work presented here, provides comprehensive corpora that contain normalized entities as well as relationships between the different entities in BEL syntax. In the next section, an overview of BEL is given. The method section describes the corpus generation for the BioCreative training and evaluation corpora as well as an additional annotated corpus resulting from BioCreative BEL track, task 2. In the result part, corpus statistics and inter-annotator agreements (IAAs) are outlined. Finally, a short overviews of the BEL track results is given.

### BEL overview

BEL is designed to represent discrete scientific findings together with their relevant contextual information as qualitative causal relationships. This formal representation ultimately facilitates knowledge-based analytics. It is to biologists what the Chemical Reaction Notations is to chemists (25). BEL uses a notation similar to the symbolic representation of a chemical reaction where the reactant entities are given on the left hand side and the product entities on the right hand side. In contrast to other systems biology modeling languages, such as BioPax (26), SBGN (27) or SMBL (28), BEL is readable and writeable by humans as well as by machines.

**Figure 1.**
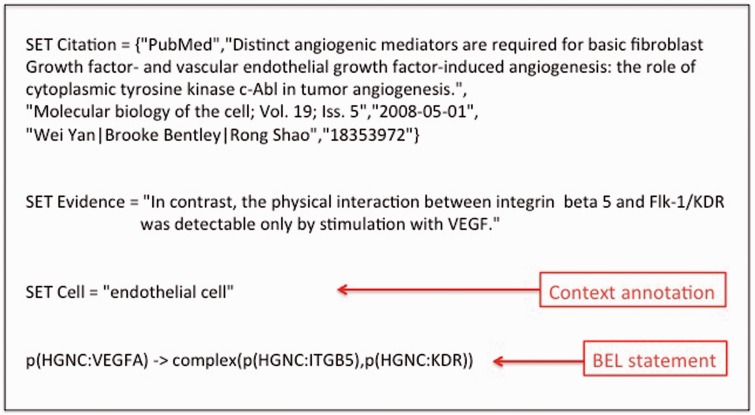
An example of a BEL statement with the associated context information.

The smallest unit of BEL information is a BEL nanopub. The concept of the nanopub is aligned to the definition of nanopublications by the Concept Web Alliance (29) (http://nanopub.org/wordpress/?page_id=65): ‘A nanopublication is the smallest unit of publishable information: an assertion that can be uniquely identified and attributed to its author’. The BEL nanopub is composed of the BEL statement that describes a causal relationship between entities. The BEL statement is also accompanied by a citation, the supporting evidence such as a text excerpt, and additional experimental context information. An example of a BEL nanopub with experimental context information is presented in Figure 1.

Composed of subject, predicate (relationship), and object triples, BEL represents biological knowledge captured in causal and correlative relationships. The language supports the collation of scientific findings to dynamically assembled network models. Large network models are considered a causal network knowledgebase, while small models represent pathways. A BEL knowledgebase can be used to query, interpret, and analyse, and can be visualized as graphs (2, 23, 30, 31). Representing relationship information in a computable format serves systems biology and toxicology approaches, especially the network-based approaches that have emerged as powerful tools for interpreting high-throughput data (1, 32–36).

One of the largest publicly available BEL-coded biological network repositories is Causal Biological Networks (CBN) (http://www.causalbionet.com/) (31). This database comprises multiple versions of >120 biological network models that have undergone extensive manual curation. The networks represent causal signaling pathways across biological processes such as cell fate, cell stress, cell proliferation, inflammation, tissue repair, and angiogenesis in the pulmonary and vascular systems. Public manual curation is facilitated by Bionet, a cloud-based crowd verification portal in the frame of the sbvIMPROVER Network Verification Challenge (32). In the first challenge, 50 network models based on human non-diseased respiratory tissue, including biological processes such as cell stress, cell fate, cell proliferation, immune, and tissue response, were provided on the Bionet website (https://bionet.sbvimprover.com/) for the challenge participation. During the sbvIMPROVER network verification challenge, participants could enhance and refine the networks and add mechanistic detail, as well as critically review existing BEL nanopubs and nanopubs added by other users. In total, 200 new nodes and 487 new edges were added by the participants. The outcome of the challenge was made available in the CBN as new versions of corresponding networks. The BEL nanopubs stored in this database together with further BEL resources provided by Selventa (www.selventa.com/science.html) served as a starting point for the generation of the BEL corpora.

OpenBEL adopts external terminologies and ontologies and uses these concepts in the BEL statements. Every concept has a unique name or identifier and refers to its entity class (also referred to as ‘namespace’ in OpenBEL). In the example given in Figure 1, all three entities VEGFA, ITGB5 and KDR reference the namespace HGNC, the database for human gene names provided by the HUGO Gene Nomenclature Committee (37). The syntax for the representation of entities is always ‘<namespace reference>:<entity reference>’. Currently, over 20 different namespaces are provided by OpenBEL for use in BEL statements, which can be found at the OpenBEL website (http://wiki.openbel.org/display/BELNA/Namespaces+Overview).

An overview of all functions and relationships used in the corpora is given in Table 1. Most have both short and long forms, which can be used interchangeably in BEL statements. In BEL terms, a namespace concept is always associated with an abundance or process function. For representing a gene, protein, or RNA, the abundance functions *geneAbundance()*, *proteinAbundance()*, and *rnaAbundance()* are used, respectively. For chemical entities, the abundance function *abundance()* is provided. Disease and biological process entities are expressed in the *pathology()* and *biologicalProcess()* functions, respectively. Post-translational modifications of proteins can be described using the *proteinModification()* function within a *proteinAbundance()*. In the published corpora, we focus only on modification by phosphorylation (*proteinModification(P)*). A number of other protein modification functions can be found in the OpenBEL language specification. The transformation functions describe the biological processes of degradation (*degradation()*) and translocation of abundances (*translocation(), cellSecretion()* and *cellSurfaceExpression()*).

**Table 1.** An overview of all functions and relationships used in the corpora

Short Form	Long form	Example	Example description
**Abundance Functions**			
**a()**	**abundance()**	a(CHEBI:water)	the abundance of water
**p()**	**proteinAbundance()**	p(HGNC:IL6)	the abundance of human IL6 protein
**complex()**	**complexAbundance()**	complex(NCH:”AP-1 Complex”)	the abundance of the AP-1 complex
		complex(p(MGI:Fos), p(MGI:Jun))	the abundance of the complex comprised of mouse Fos and Jun proteins
**composite()**	**compositeAbundance()**	composite(p(MGI:Il13),p(MGI:Ifng))	the abundance of Il13 and Ifng protein, together
**g()**	**geneAbundance()**	g(HGNC:ERBB2)	the abundance of the ERBB2 gene (DNA)
**m()**	**microRNAabundance()**	m(MGI:Mir21)	the abundance of mouse Mir21 microRNA
**r()**	**rnaAbundance()**	r(HGNC:IL6)	the abundance of human IL6 RNA
**Modification Functions**			
**pmod()**	**proteinModification()**	p(HGNC:AKT1, pmod(P))	the abundance of human AKT1 protein modified by phosphorylation
		p(MGI:Rela, pmod(A, K))	the abundance of mouse Rela protein acetylated at an unspecified lysine
		p(HGNC:HIF1A, pmod(H, N, 803))	the abundance of human HIF1A protein hydroxylated at asparagine 803
**sub()**	**substitution()**	p(HGNC:PIK3CA, sub(E, 545, K))	the abundance of the human PIK3CA protein in which glutamic acid 545 has been substituted with lysine
**trunc()**	**truncation()**	p(HGNC:ABCA1, trunc(1851))	the abundance of human ABCA1 protein that has been truncated at amino acid residue 1851 via introduction of a stop codon
**fus()**	**fusion()**	p(HGNC:BCR, fus(HGNC:JAK2, 1875, 2626))	the abundance of a fusion protein of the 5' partner BCR and 3' partner JAK2, with the breakpoint for BCR at 1875 and JAK2 at 2626
		p(HGNC:BCR, fus(HGNC:JAK2))	the abundance of a fusion protein of the 5' partner BCR and 3' partner JAK2
**Tansformation Functions**			
**deg()**	**degradation()**	deg(r(HGNC:MYC))	the degradation of human MYC RNA
**sec()**	**cellSecretion()**	sec(p(MGI:Il6))	the secretion of mouse Il6 protein
**surf()**	**cellSurfaceExpression()**	surf(p(RGD:Fas))	the cell surface expresion of Rat Fas protein
**tloc()**	**translocation()**	tloc(p(HGNC:NFE2L2), MESHCL:Cytoplasm, MESHCL:”Cell Nucleus”)	the event in which human NFE2L2 protein is translocated from the cytoplasm to the nucleus
**rxn()**	**reaction()**	rxn(reactants(a(CHEBI:"leukotriene D4")), products(a(CHEBI:"leukotriene E4")))	the reaction in which the reactant leukotriene D4 is converted into leukotriene E4
**Activity Functions**			
**act()**	**molecularActivity()**	act(p(RGD:Sod1))	The activity of rat Sod1 protein
**cat()**	**catalyticActivity()**	cat(p(RGD:Sod1))	the catalytic activity of rat Sod1 protein
**chap()**	**chaperoneActivity()**	chap(p(HGNC:CANX))	the chaperone activity of the human CANX (Calnexin) protein
**gtp()**	**gtpBoundActivity()**	gtp(p(PFH:”RAS Family”))	the GTP-bound activity of RAS Family protein
**kin()**	**kinaseActivity()**	kin(p(HGNC:CHEK1))	the kinase activity of the human protein CHEK1
**pep()**	**peptidaseActivity()**	pep(p(RGD:Ace))	the peptidase activity of the Rat angiotensin converting enzyme (ACE)
**phos()**	**phosphataseActivity()**	phos(p(HGNC:DUSP1))	the phosphatase activity of human DUSP1 protein
**ribo()**	**ribosylationActivity()**	ribo(p(HGNC:PARP1))	the ribosylation activity of human PARP1 protein
**tscript()**	**transcriptionalActivity()**	tscript(p(MGI:Trp53))	the transcriptional activity of mouse TRP53 (p53) protein
**tport()**	**transportActivity()**	tport(complex(NCH:”ENaC Complex”))	the ion transport activity of the the epithelial sodium channel (ENaC) complex
**Process Functions**			
**bp()**	**biologicalProcess()**	bp(GO:”cellular senescence”)	the biological process cellular senescence
**path()**	**pathology()**	path(MESHD: Atherosclerosis)	the pathology Atherosclerosis
**Relationship Types**			
**->**	**increases**	cat(p(MGI:Crk)) increases p(MGI:Bcar1,pmod(P))	the catalytic activ form of the mouse portein Crk induces phosphorylation of the mouse protein Bcar1
** = >**	**directlyIncreases**	cat(p(MGI:Crk)) directlyIncreases p(MGI:Bcar1,pmod(P))	the catalytic activ form of the mouse portein Crk induces phosphorylation of the mouse protein Bcar1
**-|**	**decreases**	p(HGNC:TIMP2) decreases cat(p(HGNC:MMP2))	the protein TIMP2 decreases the catalytic activity of MMP2
**=|**	**directlyDecreases**	p(HGNC:TIMP2) directlyDecreases cat(p(HGNC:MMP2))	the protein TIMP2 decreases the catalytic activity of MMP2

Many different enzyme activities for proteins and protein complexes can be expressed in BEL through activity functions. For the BioCreative evaluation, these specific activities were transformed to the general activity function *molecularActivity()*. However, the specific activity functions are retained within the corpora. All of the activity functions are briefly described in Table 1.

The relationship types found in the corpora are ‘increases’, ‘decreases’, ‘directlyIncreases’ and ‘directlyDecreases’. For a more extensive description of BEL syntax, we refer to the language specification at the OpenBEL website (http://wiki.openbel.org/display/BEL/BEL+Language+Home).

### Short overview of the BioCreative V BEL track

In the BEL track, we assessed how far automated approaches can support BEL statement extraction. Two main tasks were provided:

Task 1: Given text excerpts for a BEL statement, generate the corresponding BEL statement.

Task 2: Given a BEL statement, provide at most 10 supportive text excerpts.

For both tasks, the ***BEL_Extraction training corpus*** described in this article was provided as training data to the users. The ***BEL_Extraction training corpus*** is restricted in an automated way to the entity classes, functions, and relationships selected for the BioCreative V BEL track. In addition, for task 1, two smaller corpora were provided. The ***BEL_Extraction sample corpus*** was made available during the BioCreative task for proper system evaluation during development. For the task 1 final evaluation of the participating systems, the ***BEL_Extraction test corpus***was used. This corpus was not available to the users during the BioCreative training phase.

Since automatic extraction of BEL statements is a complex task, the BEL track provided different evaluation strategies to reduce complexity. First, the BEL statements were simplified during evaluation. These simplifications were performed only within the evaluation algorithm and not in the corpora. Thus, the users (i) got access to the BEL statements as extracted by biological experts but (ii) had the opportunity to develop systems that extract statements less complex than the original BEL statements. The first simplification concerns the organism disambiguation. In the BioCreative evaluation, orthologous genes were considered equivalent. Second, no differentiation between unspecific and direct relationship types was performed. The relationship types ‘increases’ and ‘directlyIncreases’ are treated as equal. The same is true for ‘decreases’ and ‘directlyDecreases’. Third, a system is given credit if it is able to discover any kind of molecular activities. The different BEL activity functions are all mapped to the activity function *activity()*. Finally, for the modification function *proteinModification()*, only the argument *P* is mandatory, the other arguments can be omitted. Similarly, arguments for *translocation()* function are omitted in the evaluation. In this way, the complexity is reduced within the BioCreative V BEL track evaluation but the corpus can be reused for more complex evaluations at a later stage.

The second strategy for the evaluation was to honor not only full statement prediction but also give credit for partially correct submitted BEL statements. Therefore, a cascade model was provided in the BioCreative evaluation. Term, function, relationship, and full BEL statement level evaluation scores were calculated by using precision, recall, and F-measure as evaluation metrics. This way it is possible to evaluate the capability of the systems at each level. For a more detailed overview of the BioCreative V BEL track and the evaluation results, we refer the reader to (38).

For the second task, the systems should identify supporting text excerpts from the literature for a given statement. The selected test set contains 100 BEL statements in the ***BEL_Sentence Classification Statementcorpus***. For this task, the training data was less suitable since no negative training examples were given in the corpus. The ***BEL_Extraction training corpus***contains only positive examples.

For the purpose of machine learning, not only positive but also negative examples are typically provided in this type of challenge. Those negative example text excerpts were not available for the BioCreative BEL task. However, during the task evaluation, annotations of false positive (= negative) text excerpts for a given set of BEL statements were done. To provide these positive and negative examples, we decided to publish the annotated corpora from the task 2 BioCreative evaluation as an additional corpus. Therefore, predictions of task 2 form the basis for the creation of the ***BEL_Sentence_Classification corpus.***

## Materials and methods

### Corpus selection

The starting material for the BioCreative BEL corpora was provided by the svb IMPROVER Network Verification Challenge (https://bionet.sbvimprover.com/) and Selventa. These BEL nanopubs (BEL statements along with associated citations, supporting evidence text, and context annotations) were filtered to create a ***BEL_Base corpus*** such that the constitutive BEL statements: (i) use restricted sets of namespaces, functions, and relationships for simplicity and (ii) are associated with a PubMed citation and supporting text excerpt that facilitate the training of text mining systems. The statements were mainly extracted from abstracts, but included excerpts from full-text paper as well. The supporting evidence text is derived from textual content and from tables, figures or supplementary materials included in full-text articles. Several BEL statements can be derived from a single supporting evidence source. Furthermore, additional annotations related to the context of experiments such as different disease/cell or anatomy information are also available. Hence, the BEL nanopubs can be completely identical and differ only in their context annotation information, i.e. when the text reports an observation made in several different experimental systems.

To reduce the complexity of the corpus while at the same time retaining the multimodality of the relationships, we focused on entity classes representing genes and proteins, chemical compounds, disease expressions and biological processes. Therefore, in the published corpora, we focus on the namespaces ***HGNC***for human genes, ***MGI*** for mouse genes (39), ***EGID*** for human and mouse EntrezGene identifiers (40), ***ChEBI*** for the representation of chemical entities, ***MESHD*** for diseases (41) and ***GOBP***for referencing biological processes (42).

A number of filters were applied to the initial BEL material to produce the *BEL_Base corpus*. First, all experimental context annotations were removed because only BEL statements with the citation information and supporting evidence text are of interest for the derived corpora. Second, duplicates were removed. Third, to optimize the supporting evidence text for the training of text mining systems, we selected BEL nanopubs meeting the following criteria:

- The BEL nanopub is associated with a PubMed citation.
- Supporting evidence text is associated with fewer than five BEL nanopubs in total, to avoid nanopubs derived from tables, figures, or supplementary information. In these cases, the supporting evidence text only references this information such as ‘cf. Table 1’ without any informative content.
- Supporting evidence text has a length between 36 and 425 characters. This character length was derived empirically to focus on one or two sentences.

Last, we reduced the complexity of the BEL statements. More precisely, we focused on a specific subset of entity classes, relationship types, and functions. Only statements matching the following filter criteria were considered for the training corpus:

- The statement describes the *increases*, *decreases*, *directlyIncreases*, or *directlyDecreases* relationships.
- The statement includes only HGNC, MGI, EGID, MESHD, ChEBI or GOBP namespace entities as subject or object terms.
- The statement contains no more than four named entities.
- The statement lacks the functions *compositeAbundance()* and *reaction()*.

Overall, the resulting *BEL_Base corpus* contained 29 484 unique statements from 18224 unique supporting text excerpts. For an overview of all corpora summaries we refer to Table 2. The ***BEL_Extraction training corpus*** was directly derived from this *BEL_Base corpus*. The text excerpts were randomly selected and all accompanying core BEL nanopubs were extracted. Additionally, two smaller corpora, the ***BEL_Extraction sample corpus*** and the ***BEL_Extraction test corpus***, were derived from the *BEL_Base corpus* to ensure that developers have access to properly annotated corpora for their evaluations. For the *BEL_Extraction test corpus*, we verified that the data was not publicly available elsewhere and both the *BEL_Extraction sample* and *‘test corpus’* were manually reannotated. Two senior BEL curators and a third annotator experienced in coding BEL reannotated the corpus in such a way that the supporting text excerpts contains sufficient information for the extraction of the corresponding statements and contain all possible statements for a given text excerpt. Based on the BioCreative evaluation process and participants feedback we then publish an updated version of the test and sample set. The new version of these corpora with their release notes are provided at the corpus website: http://wiki.openbel.org/display/BIOC/Datasets.

**Table 2.** An overview of all corpora summaries

Corpus	Purpose	Selection criterion	Content	No. unique sentences	No. statements
*BEL_Base corpus*	Source corpus for selection of the different corpora	Automatic filtering of expert generated BEL nanopubs	positive examples	18 224	29 484
*BEL_Extraction training corpus*	Training for task 1 and 2 BioCreative V BEL track	Randomly selected from BEL base corpus	positive examples	6353	11 066
*BEL_Extraction sample corpus*	Evaluation for task 1 during training phase BioCreative V BEL track, task1	Randomly selected from BEL base corpus; reannotated	positive examples	183	354
*BEL_Extraction test sentence corpus*	Provided during test phase BioCreative V BEL track, task 1	Randomly selected from BEL base corpus;		105	
*BEL_Extraction test corpus*	Gold standard for evaluation of BioCreative V BEL track, task 1	Randomly selected from BEL base corpus; reannotated for task 1 evaluation	positive examples	105	202
*BEL_Sentence Classification Statement corpus*	Provided during test phase BioCreative V BEL track, task 2	Randomly selected from BEL base corpus; reannotated; unpublished			100
*BEL_Sentence Classification corpus*	Annotated for future training of task 2; not available during BioCreative V	Predicted by two different systems, one prediction done by a system participating in the BioCreative BEL track task 2	positive and negative examples	806 Fully supportive: 316 TP/490 FP Partially Supportive: 429 TP/377 FP	100

### Semiautomatic corpus generation for a predefined set of BEL statements

For task 2 test set, 100 BEL statements were extracted from the *BEL_Base corpus*. First, BEL nanopubs that are not included in the other *BEL_Extraction corpora* were selected. Second, an annotator verified the correctness of the nanopubs. Third, only BEL statements were selected in which the supporting text excerpt could be found in Medline. In this way, we verified the presence of at least one text excerpt in Medline for every statement. Those BEL statements were given to the BioCreative BEL track task 2 participants as ***BEL_Sentence_Classification statement corpus*** to extract sentences from Medline abstracts as well as PubMed Central full-text articles.

**Figure 2.**
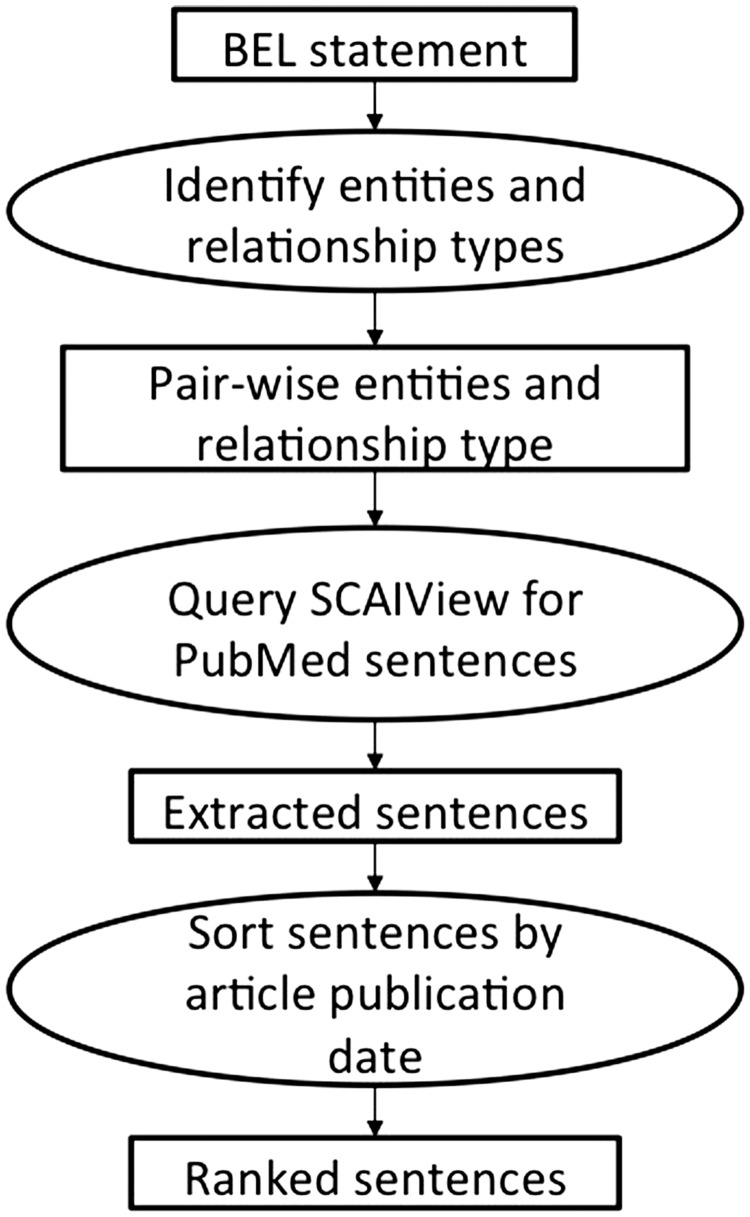
The workflow of the first method employing the semantic search engine SCAIView.
- The second method consists of two main components: a retrieval and a ranking component (see Figure 3). For an underlying BEL statement, the retrieval component identifies the sub parts of the BEL statement, adds the corresponding synonyms from dictionaries and translates everything into a search engine query. In the next step, it gathers the relevant documents from PubMed and PubMed Central from the search index. The ranking component identifies the significant supporting text excerpts and ranks them according their relevance. Similarly to the first method, up to 10 different text excerpts were evaluated as part of the *BEL_Sentence_Classification corpus*. This system was the only system participating in the BioCreative V BEL track task 2. Further details and evaluation results have been published by Rastegar-Mojarad *et al.* (44).

The predictions of task 2 form the basis for the creation of ***BEL_Sentence_Classification corpus.***Unfortunately, in the task 2 challenge, only one system participated. In order to reduce the bias towards this system, we added another set of sentences created by a simple tri-occurrence approach without further ranking methods. As a result, the *BEL_Sentence_Classification corpus* contains true and false BEL nanopubs. Both methods are described below:

- The first method can be defined as a semantic tri-occurrence approach (see Figure 2). For an underlying BEL statement, all entities and the relationship types are identified. This information is used to query PubMed sentences using the semantic search engine SCAIView API (43). SCAIView (SCAIView is available at http://www.scaiview.com/) is an information retrieval system that incorporates PubMed documents and annotations, created for various entity classes (e.g. HGNC, MGI and ChEBI) by the NER tool ProMiner, within a Lucene-based search index. The API method searches the sentences that contain the two entities and a trigger word matching the relationship type. The resulting sentences are sorted by the publication date of the article and provided to the curators. A maximum of two sentences could be proposed as a single excerpt. This size restriction was established to limit the curation workload as all submitted text excerpts had to be manually reviewed. For the *BEL_Sentence_Classification corpus*, up to 10 different pieces of text excerpts were evaluated for each BEL statement.

**Figure 3.**
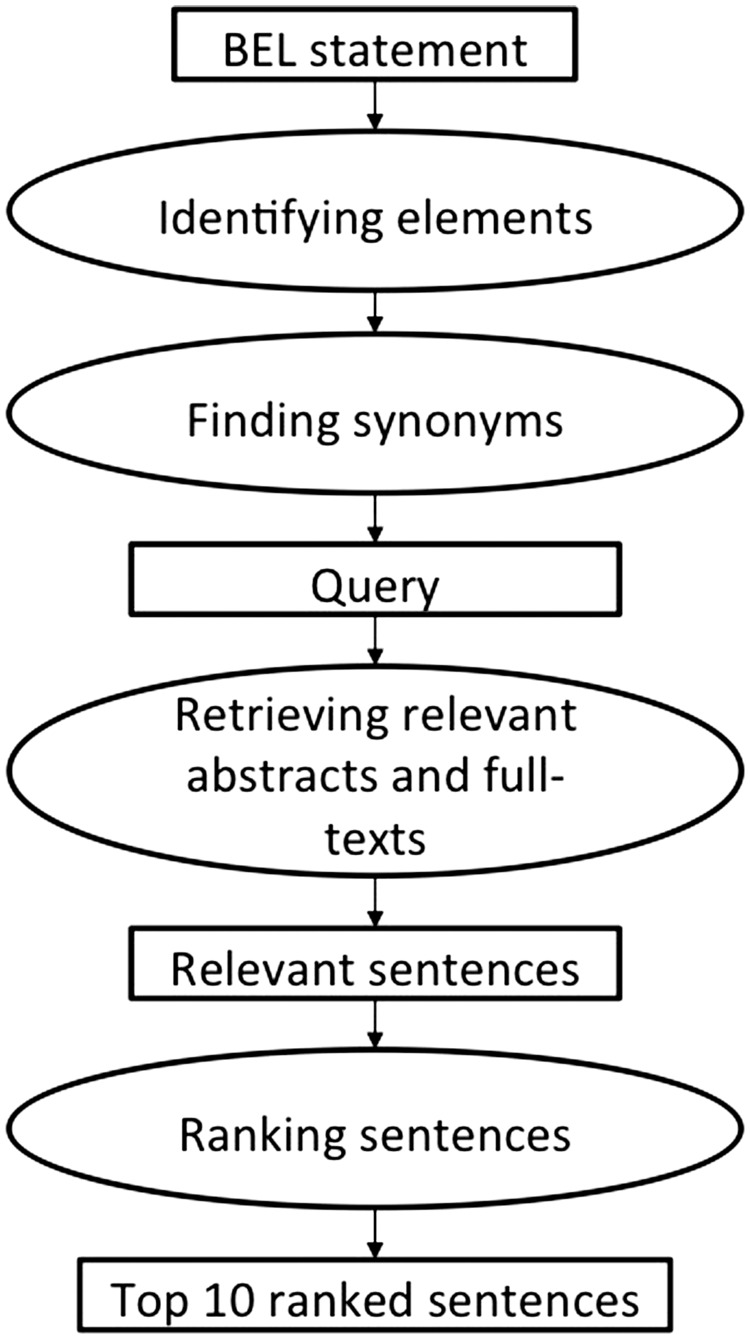
The workflow of the second method implemented by Rastegar-Mojarad *et al.* (44).

### Corpus data formats

For each *BEL_Extraction sample*, *training*, and *test corpus*, three files are provided: *.tab*, .*BEL* and .*sentence* files. The *.tab* files contain tab-separated content with the following 5 columns: BEL-ID, BEL statement, unique sentence ID, the sentence and the PMID.

**Figure 4.**
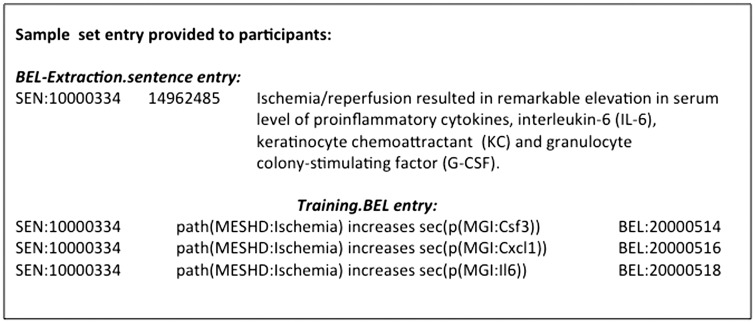
An example of the BEL_Extraction sample corpus.

The BEL-ID is a unique ID for every BEL nanopub and similarly, the sentence ID is a unique ID for every text excerpt in the corpus. The PMID is the unique PubMed accession number used as PubMed reference. The *.BEL* files contain the BEL-ID and the BEL statement. The *.sentence* files provide the sentence ID, the PMID, and the supporting evidence text. In Figure 4, an example from the sample set is given.

The *BEL_Sentence_Classification* corpus is composed of a *.tab* file containing the BEL ID, the BEL statement, the PMID, and the extracted text excerpt. The text excerpt has a maximum limit of two sentences. The next columns contain the classification label information that describes whether the sentence is fully or partly supportive for the given statement. An excerpt is defined as fully supportive (true positive) if all information concerning the BEL statement can be extracted or directly inferred. It is annotated as partly supportive (equivalent to relaxed true positive in the BioCreative V BEL track task 2 evaluation) if further contextual information or biological interpretation is required to extract the corresponding BEL statement from the given text. Annotation examples can be found in the next section.

## Annotation Guidelines

In the following, annotation guidelines for the *BEL_Extraction* corpora and the *BEL_Sentence_Classification* corpus are described.

### BEL_extraction corpora

The *BEL_Base* corpus contains BEL statements from two distinct sources, each with a different purpose and history. The set of statements from Selventa were curated over the course of more than a decade by a team of scientists. Although a generally good quality was maintained, the curation guidelines evolved significantly over this time period along with the BEL language. The sbv IMPROVER set includes statements generated in a crowd sourcing approach by scientists with a broad range of background and experience. Neither source was developed specifically for the purpose of training automated text mining applications. Due to the heterogeneity of the *BEL_Base* corpus, it is not useful to provide strict annotation guidelines describing the curation of the base corpus. Instead, we focus on detailing the best practices applied to the smaller corpora that were re-annotated to meet these strict guidelines. In parallel, we show examples of deviations observed in the provided training data.

The guidelines are based on the BEL language specification version 1.0 and describe the way in which BEL corpora are generated. For further explanations of BEL syntax, we refer the reader to the BEL language specification.

As described in ‘corpus selection’ section, an automated process selected the *BEL_Base corpus*. A subset of this corpus, the *BEL_Extraction training corpus*, was directly provided for training purposes. In the following annotation guidelines, we show examples of this corpus and provide in addition best practice advices.

For the *BEL_Extraction sample* and *test corpus*, in addition, it must be possible to extract the BEL statement from the supporting text excerpt without much background biological knowledge or biological interpretation. Otherwise, the performance of the automated systems cannot be estimated correctly. To create those corpora, we re-annotated the original statements to follow best practice annotation. Hence, BEL nanopubs qualify for the corpora only if the full statement information can be retrieved from the given supporting text. In detail, all entities and corresponding modification have to be named in the text excerpt and all functions need to be mentioned. If necessary, the given statements are corrected in such a way that only the information in the excerpt is coded.

Furthermore, for the purpose of evaluation of text mining systems that participated in BioCreative V track 4, we restrict the corpus to the namespaces and relationships that were used in BioCreative V track 4 and on BEL nanopubs extracted from scientific articles. In addition to the best practice examples, we present original BEL nanopubs and explain the changes and additions we made for the sample and test set.

For every BEL statement, the inclusion of a citation in the form of a PubMed reference is mandatory. This is not a general requirement in BEL, but is for the BioCreative task. Additionally, appropriate text excerpts supporting the BEL statement must be specified. The use of co-references is only permitted if the full entity information is given in the excerpt. A co-reference example is given in Figure 4. In this sentence, ‘Cytokines’ is a co-reference for the entity information ‘interleukin 6’, keratinocyte chemoattractant’ and ‘granulocyte stimulating factor’. Best curation practice is to select the excerpt in such a way that information about the experimental conditions is included. Alternatively, result and summary sentences clearly expressing the corresponding relationship may be chosen. For the *BEL_Extraction sample* and *test corpus*, the information within the text excerpt should be sufficient to derive the corresponding BEL statement. Moreover, the text excerpt should be no longer than two sentences. In such a way, a comparable degree of difficulty for BEL statement extraction was ensured for the BEL task.

In best practice curation, the correct biological interpretation from experimental settings is derived and the causal biological relationships are expressed in the BEL statements. Examples of such interpretations are given below (see Examples 9–16). In these examples, the named entities used in the statements ‘are underlined’ in the supportive evidence text. Further best practice examples can be found at the OpenBEL website (http://wiki.openbel.org/display/BEL/BEL+Best+Practices). Examples are also given for the cases where *sample* and *test corpus* annotations differ from best practice. In these cases, we adapted or extended the annotations to allow a more consistent evaluation.

In BEL statements, only normalized entities are used. All defined names of entities can be found in the corresponding namespace. For example, entries in the CHEBI BEL namespace are restricted to be used with the abundance function *abundance()*. Similarly, entries in gene namespaces like HGNC are restricted to *geneAbundance()*, *rnaAbundance()*, *microRNAAbundance()* and *proteinAbundance()*, with the options for specific entities restricted by the type of gene. For example, microRNA namespace values like ‘HGNC:MIR21’ can be used with *geneAbundance()*, *microRNAAbundance()*, or *rnaAbundance()*, but not *proteinAbundance()*.

If there are any special characters in the entity name, such as a space, hyphen, comma, bracket, or symbol, the name must be enclosed by quotation marks. OpenBEL provides namespaces for preferred names and the corresponding namespaces for database identifiers (IDs). To improve readability, we recommend using the preferred names instead of the IDs.

For biological processes, it is preferable to use only ‘root’ entities and not regulatory child terms. For example, *GOBP:*‘*synaptic transmission, cholinergic’* is a root entity with the regulatory children *GOBP:* ‘*regulation of synaptic transmission, cholinergic’* and *GOBP:* ‘*negative regulation of synaptic transmission, cholinergic*’. These regulatory concepts can be represented in BEL statements using the BEL relationships i*ncreases* or *decreases* together with the root entity and thus their use is best avoided.

Example 1: “Galanin” induces “cholinergic” dysfunction.

**Table:** 

Best practice:	*p(HGNC:GAL) decreases*
	*bp(GOBP:”synaptic transmission, cholinergic”).*
Not recommended:	*p(HGNC:GAL) increases*
	*bp(GOBP:‘negative regulation of synaptic transmission, cholinergic’)*

Similarly, if proteins or chemicals are involved in a biological process entity name, it is preferable to use the chemical or protein entities directly.

Example 2: In particular, “acetylcholine” release is inhibited by “galanin”.

**Table:** 

Best practice:	*p(HGNC:GAL) decreases sec(a(ChEBI:acetylcholine)).*
Not recommended:	*p(HGNC:GAL) decreases*
	*bp(GOBP:‘acetylcholine secretion’)*

In Example 2 best practice, the transformation function *sec() (secretion())* for the secretion of proteins is applied. Similarly, the translocation function *tloc()* and the degradation function *deg()* are employed. All of these transformations are expressed as transformation functions (e.g., *p(A)* induces *tloc(p(B))*) instead of using active relationships (e.g., *p(A)* translocates *p(B)* does not exist in BEL). Further best practice examples for these functions are as follows.

Example 3: When cells were treated with “brefeldin A”, we observed “STK16” translocation to the nuclear compartment.

**Table:** 

Best practice:	*a(ChEBI:‘brefeldin A’) increases tloc(p(MGI:Stk16),GOCC:cytoplasm, GOCC:nucleus)*

In addition to the named entities *ChEBI:‘brefeldin A’* and *MGI:Stk16*, the location information is defined with normalized entities as well. The GO cellular component (GOCC) namespace is used for coding the location information (For the sake of simplicity, this location information encoded in names from the GOCC namespace is not used in the BEL track evaluation. *a(ChEBI:‘brefeldin A’) increases tloc(p(MGI:Stk16))* would be fully accepted within the evaluation environment.).

Example 4: “Eukaryotic initiation factor (eIF) 4B” interacts with several components of the initiation pathway and is targeted for cleavage during “apoptosis”.

**Table:** 

Best practice:	*bp(GOBP:‘apoptotic process’) increases deg(p(HGNC:EIF4B))*

Post-translational modifications of proteins can be directly expressed in modification functions together with the normalized entity. For an overview of all possible variations of the modification terms, we refer the reader to the syntax specification.

The next example shows the minimum information required to describe a modification by phosphorylation.

Example 5: “MAPK3” is phosphorylated by “MAP2K1”.

**Table:** 

Best practice:	*p(HGNC:MAP2K1) directlyIncreases p(HGNC:MAPK3, pmod(P))*

In many cases, two or more distinct modifications are required simultaneously for a single protein activity, and neither modification alone is sufficient. In BEL language v1.0, it is not possible to model more than one protein modification. Therefore, a simple approach is to model the effect of each site separately.

Example 6: “MAPK3” must be phosphorylated at two sites, threonine 202 and tyrosine 204, to be active.

**Table:** 

Best practice:	*p(HGNC:MAPK3, pmod(P, T, 202)) = > kin(p(HGNC:MAPK3))* and *p(HGNC:MAPK3, pmod(P, Y, 204)) = > kin(p(HGNC:MAPK3))*

Activity functions are used to represent changes in the protein activity, particularly when not due to corresponding changes in the amount of the protein. In Example 6, the kinase activity function *kin()* is used because MAPK3 is a kinase that is typically activated via phosphorylation. If known, the most specific applicable BEL activity function should be used; if not, the general function *act()* can be used.

Example 7: “c-Cbl” binds to “Fyn” upon “insulin” stimulation.

**Table:** 

Best practice:	*p(HGNC:INS) increases complex(p(HGNC:FYN),p(HGNC:CBL))*

Proteins in complexes are directly expressed with the function *complex()*. Because the corpora focus on BEL statements with causal increases/decreases relationships, the complex function is only represented in BEL statements if the protein complex is part of a causal relationship. Example 7 represents regulation of protein binding using the *complex()* BEL function. In contrast, from the sentence ‘c-Cbl binds to Fyn’ no causal BEL statement could be extracted. The next example shows the use of the complex function as the statement subject term:

Example 8: These results suggest that “integrin alpha5/beta1” mediates “fibronectin”-induced epithelial cell proliferation through activation of the “EGFR’.

**Table:** 

Best practice:	*complex(p(HGNC:ITGA5),p(HGNC:ITGB1)) directlyIncreases kin(p(HGNC:EGFR))*
Sample/Test:	*p(HGNC:FN1) increases bp(GOBP:”*epithelial cell proliferation”)

The last BEL statement is not part of the original corpus because it was not relevant to the curator. For the sample and test set it is added.

Example 9: “HMG-14” phosphorylation is severely reduced or abolished in mice lacking “MSK1” (MSK1 is a synonym for *MGI:Rps6ka5*).

In Example 9, no direct interaction can be derived from the observation described in the sentence. Hence, the relationship type ‘increases’ is applied. In the following Example 10, PAK2 protein is a substrate of CASP3; the abundances of the subject and object activity BEL terms physically interact, so the relationship ‘directlyIncreases’ can be used (For the BEL task is not necessary to differentiate between ‘directlyIncreases’ and ‘increases’ relationship type—this type is automatically converted into the type ‘increases’ during evaluation.).

Example 10: “Pak2” is cleaved by “caspase 3” during apoptosis.

Best practice would be to add the additional evidence text from the source, e.g.

‘Pak2 is cleaved by caspase 3 during apoptosis, resulting in kinase activation’.

These last two examples clearly show the interpretation level that can be used in the BEL statements. For knockdown experiments, a standard inversion of relationships as shown in Example 9 is required. In this example, MSK1 knockdown mice have reduced *MGI:Hmgn1* phosphorylation. Biologist would infer from this knockdown experiment that *MGI:Rps6ka5*, when present in normal mice, is responsible for inducing *MGI:Hmgn1* phosphorylation. In the training data, often activity is added to proteins [see Example 9, *kin(p(MGI:Rps6ka5))*]. Best practice is only to add those activity functions when they are stated in the accompanied text excerpt. For further best practice examples we refer to the OpenBEL wiki pages (Further examples in BELv2.0 format: http://wiki.openbel.org/pages/viewpage.action?pageId=10388150). In re-annotation of the *BEL_Extraction sample* and *test corpus*, activity status not found in the text is removed.

In Example 10, if it is known that processing (in this case, expressed as cleavage) of a protein induces its function, the activation of the function within the BEL statement is a standard interpretation often used in the *BEl_Base* corpus. As shown above, best practice would be to include a text excerpt describing the activation, deactivation or degradation. Nevertheless, we decided to retain this interpretation level in the *BEL_Extraction sample* and *test corpus* since it is often found in the corpus.

Example 11: V12“Ras” was able to induce “cyclin D1” expression (Cyclin D1 is a synonym for *HGNC:CCND1*).

In Example 11 best practice (1), the normal protein HRAS is used because V12Ras is known to be always active and is used to investigate signaling of active ras. Therefore, the activated normal form of the protein can be used in the BEL statement. Alternatively, the relationship with the mutated variant can be expressed (see best practice (2)). The recommendation for the best practice depends on the context of the publication. The first variant should be applied if the mutated protein is used in experiments to find the normal function of active Ras. If the (unknown) function of the mutated protein is the subject of investigation, the second variant would be defined as a best practice coding. The *BEL_extraction* corpora were restricted such that they did not include protein variants (proteins modified with the sub, trunc or fus functions). Hence, sample and test corpus contain only statements similar to best practice (1).

Further biological interpretation and integration of background knowledge are allowed as far as the information is common biological knowledge. The following examples illustrate a standard biological interpretation of an inhibitor experiment:

Example 12: The PI3K(“p110alpha”) inhibitor “wortmannin” inhibited the induction of “CHOP” in a dose-dependent manner (p110alpha is a synonym for *MGI:Pik3ca* ; CHOP is a synonym for MGI:Ddit3).

In Example 12, two possibilities are given, in the first version the direct result of the observed relationship, wortwannin decreases Ddit3, is coded. Because wortmannin is a *Pik3ca*, inhibitor, the inverse relationship can be described between *Pik3ca* and *Ddit3.* Depending on the context of the text excerpt, both methods of encoding are possible. For the sample and test set, always both possibilities should be generated. The direct relationship between the inhibitor and the inhibited protein is always created as an additional BEL statement.

Example 13: “Adhesion” to fibronectin induced “PLC-gamma1” tyrosine phosphorylation that was inhibited by an “Src-kinase” inhibitor.

In Example 13, to represent the relationship between SRC and PLLG1, only the best practice interpretation is possible. Through the effect of the Src-kinase inhibitor, it became clear that the active SRC is responsible for the tyrosine phosphorylation. Therefore, the kinase activity *kin()* is used. In addition, further levels of interpretation of the text excerpt could lead to the proposed nested statements. A correct interpretation is given in the first nested statement (labeled as correct); a false interpretation is shown with the second nested statement (labeled as false). The best practice recommendation is to omit nested statements as much as possible. Hence, these nested statements are not included into the sample and test set.

Example 14: The sensitivity to “Fas”-induced “cell death” was reduced in “HGF” transfectants, which was reversed by the presence of anti-“HGF” antibody.

In Example 14, the preferred way to represent the described relationship is a nested statement. If no further information is available, the relationship should be expressed in such a way. Again, in this example the difference between an entry in the training corpus and best practice is shown. No FAS-activity can be inferred from the sentence, therefore it should be omitted. Furthermore, in the test and sample set, the simpler BEL statement should be added.

In the following, two more examples are shown that describe some limited biological interpretation and are allowed in the sample and test set. If the activation of a promoter is mentioned, it can be translated to *increases r()* or as shown in Example 15 to *directlyIncreases r().*

Example 15: Cell transfection experiments demonstrated that the promoter of the “adhesion molecule L1” is activated by “KLF7” binding to CACCC motifs (adhesion moleule L1 is a synonym for MGI:L1cam).

Similarly, if the induction of DNA binding for a transcription factor is described, the interpretation it is valid to transform that into *increases tscript()* as presented in Example 16.

Example 16: “C/EBP beta” DNA-binding activity is induced in NIH-3T3 beta 2 cells exposed to “dexamethasone” (C/EBP beta is a synonym for MGI:Cebpb).

### BEL_sentence_classification corpus

The statement—evidence text pairs from the *BEL_SentenceClassification* corpus were annotated in a similar way. In contrast to the annotation for the *BEL_Extraction corpus*, we needed to evaluate whether the text excerpts can serve as a source to extract the given BEL statement. In contrast to the guidelines for curators, the excerpt is accepted as fully supportive even if it contains hypothetical statements or ambiguous organism associations. The focus for this annotation is to select excerpts that contain all information to generate the given BEL statement.

Unfortunately, the automated systems provided the text excerpts without further information on the context of surrounding text. In the course of the evaluation, it became evident that in some cases, it is difficult to rely only on the information given in one sentence. To some extent, further context details would be required to make a more assured decision. In the BioCreative evaluation, we therefore used three different annotation levels (full relationship true/false, relaxed relationship true/false, and context relationship true/false). During the IAA measurements, we refined our annotation guidelines to avoid ambiguities in the dataset. This approach led to some modifications in the underlying annotations. For the level of context relationship annotation, we were not able to define the annotation guidelines in such a way that good IAA agreement could be achieved. Consequently, we dismissed this third annotation level that was used in the BioCreative challenge beforehand and decided to publish only the newly annotated corpus.

A relationship is annotated as ‘fully supportive’ if the BEL statement is fully expressed in the text excerpts. Such excerpt is self-sufficient and contains enough information to allow the extraction of the corresponding statements. This is the case if all entities, functions, and their relationships can be extracted directly from the excerpt. However, straightforward biological interpretations are explicitly allowed and hypothetical relationships are accepted as well.

Example 17: … such as “IGF-I”, may increase “TFF1” expression while decreasing PR levels… .

Similarly to the guideline presented earlier, examples of straightforward biological interpretation are accepted: the activation of a promoter can be interpreted as an increase of RNA expression and is annotated as ‘fully supportive’ relationship evidence text.

Example 18: However, IL-1beta was shown to inhibit the “IL-6”-induced activation of the porcine “ITIH4” promoter.

Another example is the activation of a protein. In this case, we accept sentences as being ‘fully supportive’ when the activation of a protein is mentioned and is expressed in the BEL statement as activation of the transcription factor activity *tscript()*. Similarly, in the case of antagonists, the text excerpt represents fully supportive evidence for decreasing the specific activity of the protein. Additionally, if the gene names are clearly stated and refer to orthologous genes, organism associations are ignored (see Example 18, the porcine ITIH4 promoter).

The category ‘partially supportive’ is always valid if the ‘fully supportive’ value is true. In addition to the ‘fully supportive’ level, it can also be valid if the statement can only be extracted by taking biological background knowledge or contextual details into account. However, in these cases, it should contain important information for the relationship. We introduced this level because in a ‘standard curator setting’ for BEL statement extraction, information on the context/surrounding text would be available and curators would like to find such text passages.

Example 19: The “M-CSF”-induced macrophages resulted in enhanced foam cell formation, which could be inhibited by monoclonal antibodies to “CD36” (M-CSF is a synonym for HGNC:CSF1).

Without further biological background knowledge, it cannot be decided whether the sentence represents the information given in the BEL statement. It might be that CD36 is in the path between M-CSF and the enhanced foam cell formation or that CD36 is necessary independent of M-CSF for foam cell formation. Similarly, if the relationship type is not clearly stated, only the partly supportive relationship class is valid.

Example 20: “CXCL12” secreted by human trophoblasts enhances the coordination between trophoblasts and DSCs, via the regulation of “MMP9” and MMP2.

In Example 20, it is not clear whether CXCL12 increases or decreases MMP9 and on which level (RNA, protein, or activation level) this regulation occurs. Nevertheless, the sentence contains important information for the corresponding BEL statement and should not be omitted.

Example 21: CONCLUSION: PTH can promote the bone resorption by increasing the expressions of some bone-resorbing cytokines such as OPGL, “M-CSF” and TRAIL, and then stimulating the “osteoclast differentiation” and activity.

Example 22: ‘To clarify the role of “M-CSF” in the “osteoclast differentiation”, we established a clonal stromal cell line OP6L7 capable of supporting hemopoiesis from newborn op/op mouse calvaria.

In Example 21, no relationship between *MGI:CSF1* and *GOBP:* ‘*osteoclast differentiation’* can be extracted from the sentence and therefore, it is not supportive. Although the correct relationship is described in Example 22, no result is given and therefore, it is not considered as supportive.

Example 23: IL1- and TNF-alpha-mediated stimulation of type 1 “APP” genes is synergistically enhanced by “IL6”-type cytokines.

Finally, sentences containing wrong information for the BEL statement are labeled as false for both categories. In Example 23, the sentence expresses the wrong direction and cannot be annotated as supportive for the given statement.

### IAA analysis

To measure the quality of the gold standard corpus, an analysis of the results of manual curation is performed. In an ‘IAA’ analysis, a comparison of the labels created by several curators is performed and scores are calculated that represent the agreement and also the disagreement between curators. In such a way, the quality of annotation guidelines can be assessed and indications of inconsistency in the instructions can be revealed. A high agreement score implies that the task is well defined and the corpus annotations are consistent (45). In addition, the IAA analysis gives a hint about an upper boundary for the prediction systems.

Cohen’s kappa is a popular measure that calculates the overall proportion of agreement and corrects it by taking into account the level of agreement that would occur by chance (46).

$$kappa=\frac{\left(p_{o\;}-\;p_{e}\right)}{\left(1-p_{e}\right)}$$

The overall proportion of agreement is defined as $p_{o\;}$and $p_{e}$ defines the agreement expected by chance. For the IAA calculation of the *BEL_Sentence_Classification* corpus, we calculated both, $p_{o\;}$and kappa values, for the two corpora. Landis *et al.* (47) also provide a guideline for the strength of the kappa values, which classifies the level of agreement in a range between poor and almost perfect.

For the comparison of the *BEL_Extraction sample* and *test corpus* annotations, the evaluation interface created for the BioCreative V track 4 (http://bio-eval.scai.fraunhofer.de/) [described in Rinaldi *et al.* (38)] is used. As evaluation metrics, recall, precision, and *F*-score for different structural levels (term, function and relationship) of BEL statements are generated.

The IAA are analysed for both corpora in two rounds of annotation. After the first annotation of 30 BEL nanopubs, the discrepancies between the annotators were discussed and the annotation guidelines refined. A new set of 40 BEL nanopubs is selected for new annotation and IAA calculation.

## Results

### Corpus statistics

The *BEL_Extraction training corpus* contains 6,353 sentences accompanied by 11 066 statements. The *BEL_Extraction sample corpus* is composed of 183 sentences with 354 BEL statements. Finally, the *BEL_Extraction test corpus* comprises 105 sentences and 202 statements. A summary of corpus statistics with the distributions of term, function,and relationship types is provided in Table 3. Overall, the distributions between the different classes are very similar for the three sub corpora. There is a dominant category type on each level in the training set: 87% of the terms are proteins, 69% of the functions are activations and 73% of the relationships express an ‘increases’ relationship. Similar proportions apply to the sample and test set, except for the function level, where activation covers only 46% of all cases. Under the activity function, all different enzymatic activity functions are summarized. In a large number of cases, the activity information could not be found in the corresponding text excerpts and was removed from the evaluation sets during re-annotation. Furthermore, unlike the training corpus, the *BEL_Extraction sample* and *test corpora* do not contain the functions *substitution()* and *truncation()*. For the *BEL_Extraction sample corpus*, the re-annotation changes were tracked. In the re-annotation process, overall, 35 statements (10%) were edited and 73 statements (20%) were added. Eight text excerpts and their corresponding BEL statements were removed because they did not fit into the annotation guidelines anymore.

**Table 3.** Distributions of term, function, and relationship types in the BEL_Extraction corpora

*Term* *type*	*Train*	*Sample*	*Test*	*Total*
P	19 918	497	346	20 761
A	1927	79	37	2043
bp	877	79	31	987
path	244	54	15	313
***Function type***	***Train***	***Sample***	***Test***	***Total***
act	6332	0	36	6368
pmod	1411	24	9	1444
complex	750	26	15	791
tloc	406	11	13	430
deg	205	18	6	229
sub	23	0	0	23
trunc	6	0	0	6
***Relation type***	***Train***	***Sample***	***Test***	***Total***
increases	8112	221	155	8488
decreases	2956	84	53	3093

The *BEL_Sentence_Classification corpus* includes 99 BEL statements associated with 1554 unique pieces of supporting text excerpts. A total of 578 (37%) are classified as ‘fully supportive’ and 976 text excerpts (63%) as not fully supportive (see Table 4). Under the partially supportive conditions, the true positive excerpts rise in number to 804 (52%), leaving 750 excerpts (48%) as false evidence text. Overall, 226 excerpts (15% of all excerpts) change classification from false to true when ‘partially supportive’ annotation is applied instead of ‘fully supportive’ criteria.

**Table 4.** Distribution of positive and negative sentences in the BEL_Sentence_Classification corpus

Class	True	False	Total
**Fully Supportive**	578	976	1554
**Partially Supportive**	804	750	1554

A relationship is annotated as fully supportive if the BEL statement is fully expressed in the text excerpts. It contains all information to allow the extraction of the corresponding statements

The category partially supportive is always valid if the fully supportive value is true. In addition, a relationship is annotated as partially supportive if the statement can only be extracted by taking biological background knowledge or contextual details into account.

### IAA for the BEL_extraction corpora

To calculate pair-wise IAA, the three curators annotated 40 sentences simultaneously. The BEL track evaluation interface compares two datasets of BEL statements with each other, normally a gold standard with automated prediction, and generates recall, precision, and *F*-score measures for different structural levels (term, function, and relationship) of BEL statements. For the pair-wise agreement of two curators, the first curator annotations were defined as the gold standard and the second curator dataset was treated as a prediction. Overall, the agreement between annotators one and two was very high, over 95% for most of the different levels (see Table 5). The main reason for this high agreement is that the BEL statements already exist and need only to be corrected and extended. At the statement level, the agreement was below 90% (88.89%). The reason for this is that already a single error in one of the other classes leads to an error on the full statement level. The levels of overlap with the third curator are lower, reflecting the shorter BEL coding experience of the third annotator. The main annotation differences at the term level are erroneous normalization of family names in text to gene entities such as HGNC names. For example, the term *‘IL1’* should not be mapped to *HGNC:IL1A* because it is unclear whether *IL1A* or *IL1B* is being referred to. Another kind of error can easily occur at the relationship level as shown in the next example.

**Table 5.** IAA (*F*-score) of 40 randomly chosen BEL statements from the BEL_Extraction sample corpus

Class	**Annotators**		
	**1 and 2**	**1 and 3**	**2 and 3**
**Term (T)**	97.35	94.92	95.73
**Function-secondary**	93.33	97.32	93.33
**Function**	95.24	97.48	95.24
**Relationship-secondary**	98.55	93.33	91.89
**Relationship**	97.14	86.49	89.19
**Statement**	91.18	85.33	83.78

Example 24: ‘Consistent with an involvement of this kinase, fyn-deficient keratinocytes have strongly decreased tyrosine phosphorylation levels of beta- and gamma-catenins and p120-Cas, and structural and functional abnormalities in cell adhesion similar to those caused by tyrosine kinase inhibitors’.

In Example 24, there are two correct interpretations based on the sentence syntax: the tyrosine kinase inhibitor either only causes abnormalities in cell adhesion or causes a decrease in tyrosine phosphorylation of beta- and gamma-catenins and p120-Cas as well. Consequently, different interpretation results into three different BEL statements. Overall, the agreement is very high and curator disagreement could mostly be observed in modified or added statements. In order to achieve a high overall agreement for the final *BEL_Extraction sample* and *test corpus*, a second curator checked the statements again. Discrepancies were resolved in discussion rounds with all three annotators.

### Short overview of BioCreative BEL track task 1 results

On full statement level the best system achieved an *F*-score of 0.27 when no entities are provided and 0.35, respectively, when all gold standard entities in the BEL statements are given (48). The highest *F*-score for relation extraction is 0.49 without and 0.65 with given entities. The recognition of functions and their entity assignment seems to be difficult. Even with knowledge of the correct entities, only an *F*-score of 0.3 is achieved by the best system (38).

### IAA for the BEL_sentence_classification corpus

We randomly selected 40 documents from the *BEL_Sentence_Classification* corpus to calculate the pair-wise IAA (see Table 6). The observed agreement of annotator one and two is very high with 90.0% (kappa: 0.80) for the fully supportive category and 86.7% (kappa: 0.72) for the partially supportive annotation. Similarly, between annotator one and three an agreement of 86.7%, (kappa: 0.71) for the fully supportive category and 93.3% (kappa: 0.86) for the partially supportive category could be observed. In contrast, especially for the fully supportive annotation, curator two and three has lower observed agreement of 75.0% (kappa: 0.44). For the partially supportive category, they reached 85.0% agreement (kappa: 0.687). These values were only reached after three jamboree rounds of redefining the annotation guidelines in a post-BioCreative evaluation.

**Table 6.** IAA (observed agreement and kappa score) of 30 randomly chosen entries from the BEL_Sentence_Classification corpus

Class	**Annotators**		
	**1 and 2**	**1 and 3**	**2 and 3**
Fully supportive	90.0% (Kappa: 0.80)	86.7% (Kappa: 0,72)	75.0% (Kappa: 0,44)
Partially supportive	86.7% (Kappa: 0.71)	93.3% (Kappa: 0.86)	85.0% (Kappa: 0.69)

There remain two main sources for disagreement. In the case that the annotations differ only in the fully supportive class, the annotators mostly disagree whether the sentence contain all necessary information or not. In the following such an example is given.

Example 25: Rather, these studies indicate that Egr-1 deficiency worsens liver fibrosis in conjunction with enhanced expression of the profibrogenic Itgb6 gene.

In Example 25, one annotator assigned the text as fully supportive the other one only as partially supportive. In most cases of complete disagreement, it occurs already on the level of entity normalization. In Example 26, one annotator assumed that Syn4 could be normalized to F13a1 and annotated it as supportive. The other annotator did not normalize it to that gene and didn’t annotate it as supportive at all.

Example 26: Consistent with these effects on FA dynamics and Arf6 activity, expression of huSyn4Y180L reduced migration speed following suppression of endogenous msSyn4 expression, whereas huSyn4Y180E induced directionally persistent cell migration (Supplementary Figures S4E and S4F).

These two examples show the complexity of the annotation task.

### Short overview of BioCreative BEL track task 2 results

For task 2, only one system participated during the BioCreative V BEL track task 2 assessment and provided 806 text excerpts for 96 BEL statements. Within the BioCreative evaluation, we considered three different levels of correctness: In 39.2% of the excerpts, the BEL relationship is fully expressed in the sentence, for 62.1%, the relationship can be extracted from the excerpt when context sentences or biological background knowledge are taken into account. For 72 BEL statements, there was at least one entirely correct evidence sentence, for 78 statements at least one sentence meeting the relaxed evaluation conditions (now defined as partly supportive). In comparison, under the revised annotation, the fully supportive category remains consistently at 39%, whereas the partly supportive annotation dropped from 62 to 53%.

## Discussion

The BEL corpora created for the BioCreative V track 4 add a new resource for use in the training and evaluation of biological relationship extraction methods. In comparison to other published corpora, these represent multimodal relationships spanning from molecular protein–protein or protein–chemical entity relationships to causal relationships including biological processes or diseases. There are already a number of publicly available corpora addressing different relationship types [see (17, 18, 21, 38)]. In contrast to the presented corpora, they mostly address only one or two types of entities. Furthermore, they omit the normalization of the entity classes to fixed entities. However, both the multimodality in the relationships and the normalization are needed for systems biology.

Another new aspect is that the corresponding relationships are expressed in the system biology language BEL. In such a way, automated text mining systems could directly feed into a workflow for the generation or extension of such networks. In recent years, some BEL resources have become publicly available, mainly through the CBNs database (30) and through the sbv IMPROVER NVC crowdsourcing approach (2). It would be most effective if these growing resources could directly feed into optimization processes for text mining tools. During preparation and analysis of the data, several aspects became clear for this approach. First, in a number of cases only references to tables, figures, or supplementary information are given in the BEL nanopubs as supportive evidence. Since these evidences do not express the relationship to be extracted, those nanopubs have to be removed. Second, the original corpora contain a large number of namespaces and annotation information that required a reduction of complexity. Therefore, an automated preprocessing step was included to prepare the *BEL_Extraction training corpus*. This automatically filtered corpus contains enough examples for the development and adaption of systems for BEL statement although it has certain restrictions. Curators may not strictly translate from the text excerpts and use a lot of biological interpretation that is independent from the extraction step. Hence, a fully automated BEL statement extraction is not always possible from such supporting text excerpts alone.

To cope with this restriction, for two smaller corpora, *BEL_Extraction sample* and *test corpus*, a manual re-annotation step was set up. The re-annotation efforts generated sample and test corpora where the supporting text excerpts contain sufficient information to allow the extraction of the full statement. In the cases that not all possible BEL nanopubs for the given text excerpts occurred in the source files, the missing nanopubs were added.

Together with the evaluation environment developed in the BioCreative V track 4, those two corpora were used to evaluate automated relationship extraction. In the BioCreative V BEL track, five different teams participated and used the corpora for the development of their system. The evaluation showed that the performance of fully automated BEL statement extraction is currently very low (20% *F*-score of the best system) but relationships could already be retrieved with an *F*-score of 49%. The systems did very well given the short time frame for training of complex extraction systems with multiple entity classes. In addition, the developers need to understand BEL and translate the relationships into this syntax. With the publication of these *BEL_Extraction corpora* and the availability of the evaluation environment, we hope to attract more groups in the future.

Another disadvantage of the training corpora provided for the BioCreative V BEL track was that only positive sentences were provided. Therefore, we decided to add another corpus, the *BEL_Sentence_Classification corpus.* This corpus is a result of BEL track task 2. As a starting point, 100 BEL statements were given to the participants and automatically predicted supportive sentences were submitted back by the systems. Those sentences were evaluated and now form a corpus containing supportive, partially supportive, and non-supportive sentences. In the future, this set will hopefully lead to an improvement of sentence classification as a pre-process for the extraction of BEL statements.

From the experiences in other communities, e.g. the collaboration of the organism databases in the development of Textpresso (4) or the development of PubTator (49), we can observe that these tools are adapted to the needs of users over time. In our opinion, curators and their annotation styles also need to adapt to make the derivation of training data from structured database information more straightforward. In the case of BEL resources, the first step would be to align curator and text mining guidelines further. Another step is to allow the annotation of more text mining-related provenance information as optional information within the structured data resources. This information could be easily confirmed or rejected by curators if it is predicted by an automatic system. In such a way, the amount and quality of training data would increase tremendously. Currently, the provided corpora do not contain any positional annotations of the found entities or the relationships. This is a disadvantage in comparison to the BioNLP assessments. For all BioNLP tasks, annotations are given with position information of the entities and the relationship terms but lacking the normalization information. Most machine learning algorithms that are currently available rely on this positional information. At least for the named entities, the inclusion of positional annotation would be a promising future annotation extension for the corpora. In the BEL task, the information extraction performance was enhanced significantly when the normalized named entities are given. In future steps, more in-depth annotations of entities and the relationships in the training corpora might improve the value of the corpora further.

The analysis of IAA between different curators showed that the agreement for the *BEL_Extraction* corpora was very high compared with other relationship annotations. For example, observed curator agreement for CTD reached average *F*-score levels of 85% for paper selection and 77% for correctly labeled chemical-gene/protein interactions and chemical- and gene-disease relationships (36). The reason for the high agreement in our case is that the annotators worked on a corpus that was already manually generated and only needed re-annotation. For complete new assignments of BEL statements to sentences, we can expect higher discrepancies.

In case of the *BEL_Sentence_Classification* corpus the observed agreement values were not overall satisfying. Between annotator two and three, only an agreement of 75% could be reached for the fully supportive category. Furthermore, the IAA benchmark set of 40 sentences was very low. The experience in the excerpt annotation revealed that in many cases it is not feasible to rely on the information in one sentence without further context details. For future annotations, we plan to use a more advanced curation interface, viewing the text excerpt in the context of the publication.

Furthermore, currently we annotate a text excerpt as fully supportive, if the BEL statement could be extracted from it. Database curators would restrict the fully supportive sentences even further because they consider additional features such as the experimental evidence and correct organism. Nevertheless, we believe that the corpus with its current annotation supports the selection of text excerpts for BEL statement extraction.

In future annotations with full text, we plan to add more provenance information to the corresponding BEL statements. Some BEL curators already use BELIEF (50), a curation interface using a text mining workflow to pre-annotate BEL statements (51). Annotations from this system could be directly used for training purposes if this provenance information would be available within the resulting BEL documents. This might be a feasible way to publish larger and better-annotated resources for training.

With respect to the modeling language, BEL is a living language and open to changes. In the OpenBEL consortium, a major language update is under final discussion. The next version (BELv2.0) extends and refines the initial open source release to better support the community. Additions include representation of variants at the DNA and RNA level, protein fragments, and abundance locations. One change aligned with supporting automated relationship extraction efforts is the new relationship ‘regulates’, to support cases where it is not obvious from the text if the direction of influence is increases or decreases. Another key change is the consolidation of the numerous BEL activity functions to *activity()*, since the selection of specific activity functions relies heavily on curator experience and other information outside of the text excerpts. More specific activities can be noted via a modifier term. When these BEL language changes are implemented, the corpora and annotation guidelines reported here may require updating to account for these changes. We plan to introduce new updated versions of the corpora when BELv2.0 is released.

## Conclusions

The BEL training and evaluation corpora described in this article are new resources that support the development of complex and difficult text mining tasks. However, automatic as well as manual post-processing steps are necessary for the generation of high-quality data. Better alignment of curator and annotation guidelines and more interdisciplinary work of both text miners and curators are necessary to overcome some of this additional work and will hopefully lead to better corpora and better methods supporting curation in the future. To reach this goal, more in-depth annotations and the development of pipelines for excerpt retrieval, entity recognition, entity normalization, relation extraction, semantic interpretation, and translation in standardized syntax (e.g. in form of BEL) are necessary.
